# Key features of sustainable capacities for risk communication in health emergencies: analysis of Joint External Evaluation

**DOI:** 10.7189/jogh.15.04331

**Published:** 2025-12-05

**Authors:** Asuka Takeda, Kaoruko Seino, Hiroko Okuda, Tomoya Saito, Jun Tomio

**Affiliations:** 1 Department of Health Crisis Management, National Institute of Public Health, Saitama, Japan; 2 Center for Emergency Preparedness and Response, National Institute of Infectious Diseases, Japan Institute for Health Security, Tokyo, Japan

## Abstract

**Background:**

Risk communication is a fundamental component of public health resilience during health emergencies. While the Joint External Evaluation (JEE) framework established by the World Health Organization under the International Health Regulations assesses risk communication capacity, cross-country comparisons to identify good practices that could inform improvements in global risk communication remain unexamined. We aim to identify the key elements of effective risk communication practices by analysing high-scoring countries in JEE mission reports.

**Methods:**

We conducted a retrospective observational study using publicly available JEE mission reports from 103 countries that had completed evaluations as of October 2022. Using a five-point evaluation scale, we defined good practices as highlighted strengths in risk communication for indicators reflecting ‘demonstrated capacity’ (score four) or ‘sustainable capacity’ (score five). We documented the JEE-assessed countries and score descriptions to contextualise good practice identification. We performed a cluster analysis of the extracted good practices to identify the recurring themes and key elements across five risk communication indicators.

**Results:**

We identified 420 good practices and coded them based on the JEE technical questions. Frequently cited key elements included ‘clear roles and responsibilities’, ‘regular testing and exercises’, ‘dedicated staff and funding’, and ‘feedback mechanisms from the audience’. Additionally, innovative approaches such as ‘rumour monitoring systems’ and ‘digital literacy education’ were identified, thus providing insights into practical strategies for effective risk communication during health emergencies.

**Conclusions:**

This analysis of JEE mission reports highlights the key features of sustainable risk communication capacities. By identifying key elements that can inform the development of risk communication strategies, we offer insights to help countries enhance systems and strengthen public health resilience in the face of future emergencies.

In the contemporary global landscape, effective risk communication serves as a cornerstone of public health resilience, particularly amid the growing complexity of health emergencies [1,2]. The recent emergence of pandemics has underscored the crucial role of robust risk communication strategies [3–5]. The transparency, accuracy, and timeliness of information dissemination have been essential for countries to navigate such health emergencies effectively and safeguard public health. However, during pandemics, various communication challenges have led to confusion, misinformation, and uncertainty, hampering response efforts [6,7].

These challenges are addressed within the framework of the International Health Regulations (IHR), established by the World Health Organization (WHO). The IHR mandates that all state parties develop core capacities to ‘detect, assess, notify and report events’ and ‘respond promptly and effectively to public health risks and Public Health Emergencies of International Concern’ [8]. Risk communication is a pivotal component of these core capacities. The Joint External Evaluation (JEE) is an essential initiative for building IHR core capacities, including risk communication [9,10]. This multilateral effort involves collaboration between domestic teams and external experts to assess countries’ core capacities, identify gaps in human and animal health systems, and prioritise opportunities for improvement. The JEE framework encompasses 19 technical areas, including risk communication, infectious disease response, and food safety. The JEE evaluation tool assigns scores to each indicator, enabling systematic assessment and targeted action [11].

Nevertheless, the reliability of JEE as an accurate measure of IHR core capacity is supported by the strong correlation between JEE performance and health outcome indicators in previous studies [12–15]. However, some studies have criticised its external validity and the quality of the available data sets [16,17]. For example, Japan experienced significant stigma at the onset of the COVID-19 pandemic, with risk communication ranked lowest in its JEE before the pandemic [18,19]. Conversely, Singapore communicated risk effectively during the pandemic, including emergency risk communication [20–23], and received high ratings in risk communication of its JEE before the pandemic. One study using JEE data supported the pre-pandemic hypothesis that countries with greater pandemic preparedness had more complete infection and mortality data and a lower COVID-19 disease burden [24]. The JEE scores may serve as valuable tools for identifying the gaps in essential capacities and capabilities necessary for an effective pandemic response [25]. The JEE highlights the challenges and gaps within individual countries and provides an opportunity to learn from good practices documented in other countries’ reports and apply them domestically. However, cross-country content analyses to identify patterns indicative of high and stable risk communication capacity profiles within the JEE framework have not yet been conducted. Based on the hypothesis that countries receiving high scores in risk communication in the JEE reports share common, identifiable elements in good practices – elements that are reproducible and adaptable across different national contexts and enhance risk communication capacities – we overviewed and analysed key elements across risk communication indicators in the JEE mission reports.

We aim to identify patterns indicative of high and stable risk communication capacity profiles and determine the elements that contribute to maintaining stable capacities. By recognising existing good practices from countries that scored highly in the JEE mission reports, we conducted a content analysis that considers factors such as frequency, impact, and reproducibility, to share exemplary risk communication cases. Ultimately, we seek to enhance the risk communication capacities across various countries.

## METHODS

### Data sources and selection

We used publicly available data from the WHO website. We employed the ‘JEE Dashboard’ on the WHO website to track the progress of JEE evaluations (‘Completed’, ‘In Pipeline’, or ‘Status Unknown’) by country as of 14 October 2022 [26]. For countries whose JEE status was marked as ‘Completed’, we accessed the country-specific JEE mission reports prepared by evaluators on the WHO website. We downloaded all mission reports used as data sources from the WHO website on 14 October 2022. We extracted the data on risk communication areas from these JEE mission reports. We conducted this study in accordance with the REporting of studies Conducted using Observational Routinely-collected Data guidelines [27].

### Patient and public involvement statement

Patients and members of the public were not involved in the design, conduct, reporting, or dissemination of this study.

### Descriptions of scores in JEE mission reports

Each JEE indicator is scored on a five-point scale: one indicates no applicable competence, two limited capacity, three developed capacity, four demonstrated capacity, and five sustainable capacity. According to the first edition of the evaluation tool (E1), the area of risk communication was assessed using five indicators: R5.1 Risk communication systems (plans, mechanisms, *etc.*), R5.2 Internal and inter-partner communication and coordination, R5.3 Public communication, R5.4 Involvement in communication in areas affected by emergencies, and R5.5 Vigilant listening and rumour management. We evaluated the included JEE missions using the E1 and second edition (E2) evaluation tools, while we did not use the third edition (E3). Between E1 and E2, in risk communication, the indicator names were revised; however, their attributes underwent only minor modifications, and the output and outcome indicators remained unchanged [28].

We compiled a table listing the countries whose JEE progress was marked as ‘Completed’ and for which mission reports were available. This table describes the WHO region, the year of evaluation, and the evaluation tool used (E1 or E2). From each country’s JEE mission report, we extracted and recorded the scores for each of the five indicators (R5.1–R5.5) in risk communication, along with the total score (out of 25).

We compared the scores for the five indicators in each country across the two editions of the evaluation tool (E1 and E2), categorising them into three groups: five, four, and less than three. We then calculated the number of countries in each category.

### Extraction of keywords from the technical questions

The evaluation tools (both E1 and E2) contain technical questions for each indicator (Table S1 in the **Online Supplementary Document**), which form the basis for the evaluator’s assessment.

The authors selected keywords for each indicator separately, based on both their frequency and their impact on the technical questions. These keywords were then used as the coding framework for the cluster analysis. For indicator R5.1, the selected keywords were ‘response plan’, ‘permanent and surge staff’, ‘roles and responsibilities’, ‘shared communication plans and standard operating procedures (SOPs)’, ‘financial resources for emergencies’, ‘regular testing and training’, and ‘messaging clearance’. For indicator R5.2, the keywords were ‘coordination of communication’, ‘stakeholders and partner agencies’, ‘consistency of information’, ‘formal mechanisms’, ‘coordination with the health care sector’, ‘coordination with civil society organisations’, and ‘testing communication coordination exercises’. For indicator R5.3, the extracted keywords were ‘public communication’, ‘media and social media outreach’, ‘target audience analysis’, ‘training public spokesperson’, ‘media research’, ‘message adaptation’, and ‘regular media briefings’. For indicator R5.4, the keywords included ‘social mobilisation’, ‘community engagement’, ‘health promotion’, ‘intermediate level functions’, ‘audience feedback’, ‘scaling up capacity’, and ‘baseline social data’. For indicator R5.5, the keywords were ‘rumours and misinformation’, ‘monitoring effectiveness’, ‘feedback loop’, ‘public health issues’, ‘message consistency’, ‘evaluation of communication response’, and ‘behavioural change’.

### Definition of good practice

We defined a good practice as an evaluator comment highlighted as a strength for a risk communication indicator in the JEE mission reports, associated with an indicator that received a score of four or five. The mission reports include evaluator comments from external experts on the strengths and challenges for each indicator. Multiple comments may identify strengths for a single indicator, and each comment is counted as one instance of good practice. For example, in Japan’s mission report, indicator R5.2 received a score of four and was accompanied by three comments identifying its strengths. In this case, we counted three good practices.

### Content analysis for good practices and extraction of key elements

We analysed the content of good practices using cluster analysis, with each good practice serving as a unit [29]. We performed the hierarchical clustering of textual data using NVivo, version 14 (Lumivero, Denver, Colorado, USA) clustering function. An initial keyword list, extracted from the technical questions, served as the coding framework for the clustering process. The algorithm grouped good practices by indicator based on keyword co-occurrence and textual similarity within nodes, as defined by the predefined coding framework. We excluded good practices that did not match the keywords. To ensure comprehensiveness, objectivity, and validity while minimising subjective bias, the authors independently applied the clustering process. After refinement through cluster analysis, we counted the number of good practices for each keyword. Through the clustered data, we extracted key elements for each indicator. The authors selected these key elements separately for each indicator, with particular attention to their frequency and impact in the context of good practices.

To validate the clustering results and ensure reliability, two independent experts with backgrounds in risk communication and JEE evaluations reviewed the classification outcomes. While some differences emerged in their initial clustering judgments, the overall coding patterns were highly consistent. Any discrepancies were discussed and resolved through consensus to further minimise subjectivity.

## RESULTS

As of 14 October 2022, 115 countries had a JEE progress status of ‘Completed’. Of these, 103 countries had published mission reports (**Table 1**). Countries in the African region had the highest number of JEE missions among the WHO regions. The most common year for JEE implementation was 2017, with 40 countries (38.8%), followed by 2016, with 26 countries (25.2%). E1 was used to evaluate 81 countries (78.6%), while 22 countries (21.4%) were evaluated using E2. The transition from E1 to E2 occurred in 2018.

**Table 1:** Countries assessed at least once by the JEE*

	All (n = 103)	E1 (n = 81)	E2 (n = 22)
**WHO region**			
Africa	44 (42.7)	37 (45.7)	7 (31.8)
Americas	2 (1.9)	2 (2.5)	0 (0.0)
Eastern Mediterranean	17 (16.5)	16 (19.8)	1 (6.7)
Europe	17 (16.5)	10 (12.3)	7 (50.0)
South-East Asia	8 (7.8)	7 (8.6)	1 (7.1)
Western Pacific	15 (14.6)	9 (11.1)	6 (21.4)
**Evaluated year**			
2016	26 (25.2)	26 (32.1)	0 (0.0)
2017	40 (38.8)	40 (49.4)	0 (0.0)
2018	22 (21.4)	15 (18.5)	7 (31.8)
2019	15 (14.6)	0 (0.0)	15 (68.2)

JEE – Joint External Evaluation, E1 – the first edition, E2 – the second edition

*All values are presented as numbers (percentages).

The differences in score distribution between E1 and E2 for each indicator (**Table 2**). For R5.1, the score distribution remained relatively consistent across both editions. However, for the other indicators (R5.2–R5.4), the E2 evaluation recorded fewer full scores compared to E1. Notably, in R5.4, no country received a perfect score of five in either E1 or E2; however, the proportions of scores of four were 21.0% for E1 and 13.6% for E2.

**Table 2:** Scores of the five areas of risk communication indicators from the two different editions of the JEE tools

	Countries, n (%)*		
	**All (n = 103)**	**E1 (n = 81)**	**E2 (n = 22)**
**R5.1 Risk communication systems (plans, mechanisms, *etc.*)**			
5	5 (4.9)	4 (4.9)	1 (4.6)
4	14 (13.6)	11 (13.6)	3 (13.6)
≤3	84 (81.6)	66 (81.5)	18 (81.8)
**R5.2 Internal and inter-partner communication and coordination**			
5	9 (8.7)	8 (9.9)	1 (4.5)
4	18 (17.5)	13 (16.0)	5 (22.7)
≤3	76 (73.8)	60 (74.1)	16 (72.7)
**R5.3 Public communication**			
5	8 (7.8)	8 (9.9)	0 (0.0)
4	26 (25.2)	20 (24.7)	6 (27.3)
≤3	69 (67.0)	53 (65.4)	16 (72.7)
**R5.4 Involvement in communication in areas affected by emergencies**			
5	0 (0.0)	0 (0.0)	0 (0.0)
4	20 (19.4)	17 (21.0)	3 (13.6)
≤3	83 (80.6)	64 (79.0)	19 (86.4)
**R5.5 Vigilant listening and rumour management**			
5	6 (5.8)	6 (7.4)	0 (0.0)
4	14 (13.6)	11 (13.6)	3 (13.6)
≤3	83 (80.6)	64 (79.0)	19 (86.4)

JEE – Joint External Evaluation, E1 – the first edition, E2 – the second edition

*Excludes countries where no report has been published.

We analysed the structure of good practices using a coding process based on keywords extracted from the technical questions in the JEE. We identified 420 good practices (84 for R5.1, 90 for R5.2, 129 for R5.3, 64 for R5.4, and 53 for R5.5) and categorised them based on similar or identical types using the extracted keywords (**Table 3**).

**Table 3:** Key elements selected from good practices in each indicator of risk communication*

Indicator	Number of good practices by indicator	Keyword selected from technical questions	Number of selected good practices by keyword	Key elements selected from good practices
R5.1 Risk communication systems (plans, mechanisms, *etc.*)	84	1. Response plan	10	Integration of RC into national plans; Clear roles and responsibilities; Regular review and revision
		2. Permanent and surge staff	16	Dedicated and trained staff; Sufficient resources and funding; Cross-sector collaboration and flexibility
		3. Roles and responsibilities	5	Clearly defined roles; Clear lines of responsibility; Lead agency coordination
		4. Shared communication plans and SOPs	7	Regular testing and drills; Cross-sector coordination; Updating based on real-life events
		5. Financial resources for emergencies	8	Dedicated funding for communication; Access to surge capacity; Ongoing budget for emergency situations
		6. Regular testing and training	13	Systematic testing through exercises; Continuous training for staff; Application of lessons learned
		7. Messaging clearance	6	Pre-developed templates and tools; Key message documents; Targeted communication channels
R5.2 Internal and inter-partner communication and coordination	90	1. Coordination of communication	15	Regular testing and exercises; Defined roles and structured networks; Multi-sectoral meetings and joint plans
		2. Stakeholders and partner agencies	12	Regular drills and testing with partners; Standardised mechanisms for coordination; Multi-sectoral engagement and joint planning
		3. Consistency of information	5	Unified communication networks; Collaborative messaging with international organisations; Formalised procedures for consistent communication
		4. Formal mechanisms	8	National response framework and agreements; Defined roles and responsibilities in national SOPs; RC structure across government levels
		5. Coordination with the health care sector	4	Centralised communication through EOCs; MOH as the communication hub for networks; Formal and informal mechanisms for stakeholders
		6. Coordination with civil society organisations	4	Formal mechanism for coordination; Inclusion of NGOs in emergency communication; Use of formal and informal mechanisms for stakeholders
		7. Testing communication coordination exercises	6	Annual testing with partner organisations; Active engagement of diverse partners in drills; Application of lessons learned to strengthen capacity
R5.3 Public communication	129	1. Public communication	6	Comprehensive use of multiple communication channels; Collaborative arrangements with media; Preparedness and pre-emergency campaigns
		2. Media and social media outreach	7	Multimedia platform use; Continuous public engagement; Audience-centric messaging
		3. Target audience analysis	4	Community partnerships for understanding of needs; Language and cultural relevance; Audience feedback regarding whether the messages reach them
		4. Training public spokespersons	6	Clear message delivery; Media relations and trust; Training across government levels
		5. Media research	4	Pre- and post-campaign evaluation; Collaborative media partnerships; Audience reach studies
		6. Message adaptation	5	Language relevance for diverse audiences; Cultural sensitivity for different communities; Continuous translation and local adaptation
		7. Regular media briefings	4	Scheduled regularity in media briefings; Clear communication in a timely and appropriate language; Comprehensive outreach during media briefings
R5.4 Involvement in communication in areas affected by emergencies	64	1. Social mobilisation	6	Community volunteer engagement; People/public/private partnerships (PPPP); Flexible, ad hoc teams
		2. Community engagement	9	Collaboration with community leaders and organisations; Multi-level coordination and activities; Feedback mechanisms from community
		3. Health promotion	7	Multi-level coordination; Continuous training and skill updates; Routine and emergency health promotion
		4. Intermediate level functions	9	Coordinated health promotion and education; Community-based representation and networking; Cross-sector and stakeholder collaboration
		5. Audience feedback	7	Rapid response against public reactions and questions; Feedback based refinement to improve communication; Public opinion monitoring using opinion polls
		6. Scaling up capacity	5	Flexible staffing for emergencies; Operational mechanisms for scaling; Integration into national plans
		7. Baseline social data	2	Trust and channel preferences survey; Functional feedback loop
R5.5 Vigilant listening and rumour management	53	1. Rumours and misinformation	11	Formal systems to monitor and report rumours; Rapid response capabilities with decision-makers; Public communication tools (*e.g.* apps, social media)
		2. Monitoring effectiveness	7	Regular monitoring of media (traditional and social media); Clear communication strategy; SOPs on how to address rumours, misinformation
		3. Feedback loop	5	Coordination mechanism among all relevant stakeholders; Effective communication channels; Community engagement and verification
		4. Public health issues	4	Behaviour change and lessons learned; Authoritative health advice; Digital literacy and communication
		5. Message consistency	3	Clear reporting and information checking; Public trust in health advice; Monitoring and addressing information gaps
		6. Evaluation of communication response	4	Regular evaluation of communication impact; Collaborative evaluation process; Comprehensive evaluation of communication effectiveness
		7. Behavioural change	4	Learning from past events; Systematic approach to addressing misinformation

EOC – emergency operations centre, MOH – ministry of health, NGO – non-governmental oganisation, RC – risk communication, SOP – standard operating procedure

*See Table S2 in the **Online Supplementary Document** for references.

Regarding the key elements of risk communication, for each indicator, key elements were identified based on the frequency and impact of the coded data. Across all five indicators, ‘clear roles and responsibilities’ (R5.1, R5.2) was consistently emphasised, ensuring defined task allocation and effective coordination during health emergencies. ‘Regular testing and exercises’ (R5.1, R5.2) were also frequently observed, serving as a mechanism to evaluate and strengthen communication systems. The deployment of ‘dedicated and trained staff’ (R5.1) and allocation of ‘sufficient resources and funding for communication’ (R5.1) were repeatedly cited as essential for sustaining operational readiness and enhancing institutional capacities. Several countries incorporated ‘feedback mechanisms from the audience’ (R5.3, R5.4) to verify message reach and adjust communication strategies based on recipient responses.

In addition to these widely shared practices, the analysis identified unique approaches in certain countries (Table S2 in the **Online Supplementary Document**). The establishment of ‘Public/Private/People Partnerships (PPPP)’ (R5.4) was noted in a few reports, facilitating decentralised communication networks and enabling community-level engagement for rapid information dissemination. For managing rumours and misinformation, some countries implemented ‘formal systems to monitor and report rumours’ (R5.5) through established SOPs. A few countries also introduced ‘digital literacy education’ (R5.5) programmes to enhance public capacity to assess information sources critically.

## DISCUSSION

We identified patterns of high, stable risk communication capacity profiles using JEE mission reports and derived key elements that contribute to maintaining stable capacity. The good practices associated with high JEE scores in risk communication share common characteristics, including well-defined communication structures, dedicated personnel, and consistent financial support. These findings align with previous studies that emphasise the importance of developing and maintaining sustainable systems and resource allocation for effective public health practice [12–15,30]. None of the countries achieved a perfect score of five for the R5.4 indicator (Involvement in communication in areas affected by emergencies), suggesting that community engagement during emergencies remains a global challenge. The comparison of score distributions between E1 and E2 suggests that achieving high scores became more challenging for indicators R5.2–R5.4 in E2 than in E1. This aligns with prior Delphi study findings, where 33% of respondents indicated that risk communication indicators became harder to achieve in E2, while 67% considered them unchanged [31].

The findings indicate that countries with high and stable risk communication capacities consistently share core structural key elements such as ‘clear roles and responsibilities’ (R5.1, R5.2), ‘dedicated and trained staff’ (R5.1), and ‘regular testing and exercises’ (R5.2), reflecting the importance of embedding risk communication within national governance systems. In addition, innovative practices such as ‘Public/Private/People Partnerships (PPPP)’ (R5.4) and ‘audience feedback mechanisms’ (R5.3, R5.4) highlight the value of decentralised and community-oriented strategies in enhancing responsiveness and trust. Furthermore, the proactive management of misinformation through systems such as SOP-based rumour tracking (R5.5) has become increasingly vital in today’s digital landscape. WHO’s guidelines have indicated that building sustainable and effective risk communication systems requires a combination of robust structural foundations and adaptive, community-engaged approaches [32,33]. For countries seeking to strengthen their risk communication capacities, it is essential not only to adopt these core structural practices but also to explore innovative methods tailored to their specific sociocultural contexts.

The key elements we identified through the content analysis of good practices were often aligned with the WHO guidelines for emergency risk communication published in 2018 [33]. A major distinction between WHO’s guidelines and our findings is that, while the guidelines provide a methodological framework based on a systematic review of various theoretical studies, this study compiles actual high-scoring practices from a single evaluation initiative – the JEE. We offer new insights by categorising practical risk communication strategies within the IHR framework. Countries with weaknesses or low-scoring areas in risk communication can refer to Table 3, which categorises these aspects to guide future planning and improve systems. Additionally, Table S2 in the **Online Supplementary Document** lists countries that have implemented good practices in risk communication, offering insights into factors such as national health systems and population size.

Among the countries we identified as implementing multiple good practices, Canada and Switzerland exemplified structured and responsive risk communication during the COVID-19 pandemic [34,35]. Canada established a multi-channel approach early on, using daily briefings, social media, mobile apps, and targeted outreach to Indigenous communities. Messaging emphasised anti-stigma narratives, countermeasures against misinformation, and clear guidance for high-risk groups, particularly in long-term care. Similarly, Switzerland launched a nationwide hygiene campaign immediately after detecting its first case, using multilingual materials across traditional and digital platforms. The Swiss Federal Office of Public Health held regular press briefings and addressed emerging social risks, such as domestic violence, through coordinated public information campaigns. The pre-pandemic good practices identified in the JEE are presented in Table S2 in the **Online Supplementary Document**, demonstrating how these capabilities were applied in real-world COVID-19 responses.

Future research should focus on in-depth qualitative studies to examine the contextual factors that influence the effectiveness of risk communication during emergencies across countries. Additionally, developing standardised good-practice guidelines based on the experiences of countries with high JEE scores could facilitate global knowledge sharing and capacity-building. A possible next step could involve creating step-by-step guidelines for emergency risk communication by thoroughly analysing the systems and policies of the countries with best practices and identifying commonalities among them. Furthermore, while this study primarily relied on JEE mission reports, future research should triangulate with additional data sources such as policy documents, response metrics, and interviews with in-country implementers to enhance the comprehensiveness and contextual accuracy of the findings.

Despite the valuable insights we provided in this study, some limitations should be acknowledged. First, not all WHO member states had undergone JEE at the time of this study, which limited the comprehensiveness of the analysis. Second, we relied solely on publicly available JEE mission reports, which may not fully capture the nuances of risk communication implementation across countries. Third, the variations in evaluation methodologies and reporting styles across countries may have introduced biases in the assessment of risk communication capacities. However, such bias is partly mitigated using technical questions and scoring guides provided in the JEE tool, which aim to standardise evaluations across countries. JEE’s reliance on experts primarily from high-income countries may lead to groupthink, limiting the consideration of country-specific contexts and reinforcing a rigid evaluation framework [36]. Differences in interpreting what constitutes a ‘strength’, as well as variability in how the ‘strengths’ section is utilised across different mission teams or evaluators, may affect the consistency and reliability of the findings. While the clustering and coding process aimed to mitigate this variability, these inherent subjectivities remain limitations of the current study. Fourth, the analysis did not account for contextual factors, such as political stability, socioeconomic conditions, and cultural differences, which may influence the effectiveness of risk communication. Finally, as all evaluations were conducted before the COVID-19 pandemic, we did not account for the advancements in risk communication strategies developed in response to the pandemic. However, although the evaluations we included were all conducted before the COVID-19 pandemic, the identified key elements remain relevant to current and future public health emergencies.

## CONCLUSIONS

We provided an overview of good practices in risk communication documented in the JEE mission reports and identified key elements associated with risk communication indicators. These key elements provide a framework adaptable to specific health systems, enabling countries to prioritise sustainable capacity-building and address critical challenges. The findings also offer valuable insights for policymakers seeking to understand both global and country-level contexts for the risk communication capacities required under the IHR, although further research is needed to assess the real-world effectiveness of these capacities.

## Additional material


Online Supplementary Document
