# Supplementary material for: Key features of sustainable capacities for risk communication in health emergencies: analysis of Joint External Evaluation
Source: J Glob Health. 2025 Dec 5;15:04331. doi: 10.7189/jogh.15.04331 (PMC12677237; doi:10.7189/jogh.15.04331)
Supplement: Online Supplementary Document [file jogh-15-04331-s001.pdf]

Supplement to: Takeda A, Seino K, Okuda H, Saito T, Tomio J. Key features of sustainable capacities for risk communication in health emergencies: analysis of Joint External Evaluation. J Glob Health. 2025;15:04331.

Supplemental material

Appendix Table 1. Technical questions in the E1 and E2 evaluation tools from the Risk Communication (RC) area of the Joint External Evaluation (JEE).

Tool Edition	Question No.	Technical questions by indicator
R5.1 Risk communication systems (plans, mechanisms, etc.)		
	1	Is there a function for risk communication in your national response plan?
	2	Are there communications personnel or government departments that informally respond to public information needs during emergencies?
	3	Is there a permanent or surge staff dedicated to risk communication during emergencies?
	4	Are the roles and responsibilities of the risk communication staff articulated in a response plan?
	5	Are there significant improvements that could be made in the staffing, platforms, financial resources or other factors to improve communications with the public and partners during emergencies?
	6	Are there shared communication plans, agreements and/or standard operating procedures between other response agencies such as public safety, law enforcement, hospitals, emergency response, Red Cross/Crescent and/or government agencies such as ministries of defence, agriculture, food/drug, etc.?
E1	7	Is there a dedicated budget line for communications personnel, materials and activities for emergencies?
	8	Does communication to the public during an emergency automatically revert to another government agency besides or in conjunction with the Ministry of Health?
	9	Are the plans tested on at least a yearly basis?
	10	Is training provided to the risk communications personnel for response to local hazards?
	11	Is there an agreement internal to your agency for clearance of messaging to the public?
	12	Have alterations been made to response plans based on lessons learnt from exercises or actual responses?
	13	Have communications response staff been made aware of and/or trained on response plan alterations?
	14	Is there a dedicated budget for the communications system to be sustained and to grow?
E2		Questions 8, 12, and 13 were removed from E1 and the following were added;

	15	Which government entities/agencies have the lead for risk communication for different types and magnitudes of emergencies?
R5.2 Internal and inter-partner communication and coordination		
E1	1	Is there a mechanism informally or formally to coordinate communication internal to your agency during an emergency?
	2	Is there any mechanism informally or formally to coordinate communication among national stakeholders and response agencies during an emergency?
	3	Is there a mechanism informally or formally to coordinate communication among international stakeholders and response agencies during an emergency?
	4	Have there been incidents where stakeholder/partner agencies have released information that was inconsistent or contradicted your agency's information during an emergency?
	5	Have there been incidents where valuable time was taken because of a lack of agreement regarding which agency would respond during an emergency?
	6	Do you have an example of an emergency or event that could have been better coordinated between partner agencies?
	7	Is there a formal mechanism to coordinate communication with the hospital and healthcare sector during an emergency?
	8	Is there a formal mechanism to coordinate communication among civil society organizations during an emergency?
	9	Is there a formal mechanism to coordinate communication with the private sector during an emergency?
	10	Has your organization conducted exercise testing communication coordination with partner organizations?
	11	Has your organization responded in an actual emergency that tested communication coordination with partner organizations?
	12	Does your organization regularly develop communication response plans together with external partner and stakeholders?
	13	Does your organization have a coordinated budget for communications response with external partners and stakeholders?
E2		Questions 6 was removed from E1.
R5.3 Public communication		
E1	1	Does your organization have a formalized function to communicate with the public?
	2	Does your organization have a designated and trained public spokesperson?
	3	Does your organization have a communication team dedicated to media and social media outreach?

	4	Do your organization conduct target audience analyses to better understand audience language, trusted information resources and preferred communication channels?
	5	Does your organization have a communication strategy that proactively reaches out to a variety of media platforms such as newspapers, radio, TV, social media web in order to target communication messages to specific audiences?
	6	Does your organization provide information in local languages as needed by the audience?
	7	Does your organization conduct media research to determine message reach among target audience members?
	8	Does your organization alter public health messaging according to geographic location, language and media preference?
	9	During emergencies or exercises, does your organization provide regular media briefings and updates through mass and social media?
	10	Does your organization contribute to an evidence base of what communications methods best enabled target audiences to change behaviour during emergencies?
	11	Does your organization share experience and new strategies with partner organizations to continually improve communication response?
	12	Does your organization monitor for rumours and misinformation and when found address the issues rapidly?
E2		Questions 1, 9, and 11 were removed from E1 and the following were added;
	13	Is there a fast-track process for clearing media and social media products?
R5.4 Involvement in communication in areas affected by emergencies		
	1	Does your organization have a social mobilization, health promotion or community engagement department or working group that is used for communication response during emergencies?
	2	Does your organization have a social mobilization, health promotion or community engagement department or working group that regularly works with a media department or focal person within your organization?
	3	Does your organization have a social mobilization, health promotion or community engagement department or working group that reaches out to the affected or at risk populations during health emergencies?
E1	4	Is social mobilization, health promotion or community engagement included in the national response plan?
	5	Does your organization have a social mobilization, health promotion or community engagement functions working at intermediate (district/provincial) levels?
	6	Do intermediate (district/provincial) level community engagement functions work in vertical fashion that enables national level leadership to both learn from intermediate levels and share lessons learned with other intermediate levels?
	7	Do community outreach programs regularly conduct information education communication (IEC) materials testing with members of the target audience?

	8	Does your organization regularly provide information sharing or training opportunities between experienced community engagement experts and volunteers or potential surge capacity to be used during emergencies?
	9	Does your organization have a plan to scale up existing community engagement capacities to be deployed during emergencies?
	10	Is there an ongoing and functioning feedback loop between at-risk or affected populations and response agencies?
	11	Does your organization regularly and rapidly change messaging to address audience feedback, misinformation and questions?
	12	During the last actual emergency or exercise was there a clear function to receive audience feedback or questions?
E2		Questions 5, 6, 7, 9, 11 and 12 were removed from E1 and the following were added;
	13	Are baseline social data, intelligence and analysis on factors that may increase the population's risk to (or the ability to withstand) the top five hazards in the country (such as mapping of languages, living conditions, religious/cultural practices/trusted channels of communication, influencers) conducted or commissioned?
R5.5 Vigilant listening and rumor management		
E1	1	Does your organization have a formal communication function to monitor and address rumours and misinformation?
	2	Does your organization have ad hoc methods in which to hear about some rumours regarding public health issues (health care workers, hotline information, etc.)?
	3	Does your organization have a method for addressing rumours and misinformation?
	4	Does your organization monitor the effectiveness of methods or messages used to disprove a rumour or correct misinformation?
	5	Does your organization regularly collect rumours and misinformation, the methods and messages to address them, and share them with partners to ensure message consistency?
	6	Does your organization consider communication feedback including rumours and misinformation from the public in its decision-making process to improve communication response?
	7	Does your organization regularly evaluate its communication response and ability to address rumours and misinformation to determine that actions changed behaviour and/or stopped the rumour from spreading?
E2		Questions 2, 3 and 4 were summalized from E1 and the following were added;
	8	Is the effectiveness of public outreach methods and/or messages used to address unfounded beliefs or to correct misinformation monitored?

Appendix Table 2. List of good practices with the country and reference number.

Reference No.	Keyword	Good practice	Country
R5.1 Risk communication systems (plans, mechanisms, etc.)			
1-1-1	Response Plan	A function for risk communication exists through the Media and Public Communication Emergency Supporting Plan in NCEMA, the Media Response Plan in MHP, as well as in local health authorities.	UAE
1-1-2		Regulated system of risk communication included in each emergency response plan with responsible focal points at national, regional and local levels.	Armenia
1-1-3		Risk communication is embedded in national strategies and response plans and is a key focus area of MySED II.	Malaysia
1-1-4		The MOH understands the importance of the risk communication strategy for adequate response and preparedness.	Oman
1-1-5		Risk communication is integrated into multiple communicable disease and emergency response plans.	Australia
1-1-6		Roles and responsibilities for risk communication are included in emergency response plans.	Canada
1-1-7		Thailand has integrated risk communication and public relations into plans at all levels including the National Disaster Prevention and Mitigation Plan.	Thailand
1-1-8		Risk communication response plans are reviewed and revised according to lessons learnt with external partners and stakeholders.	Thailand
1-1-9		Risk communication for health emergencies is prominent within the response structure, including in the RAG and RMG, and in the NCC communication sub-committee.	Belgium
1-1-10		The National Strategy for Disaster Risk Reduction of the Republic of Tajikistan for 2019–2030 in concert with the Unified State System on Preparedness and Response to Emergency Situations define emergency communication and coordination procedures within the government and with the public.	Tajikistan
1-2-1	Permanent and Surge Staff	Permanent and surge staff are dedicated to risk communication during emergencies. Medical and health-care professionals are defined by MHP and local health authorities (HAAD, DHA) to respond to public questions and inquiries about risks during emergencies.	UAE
1-2-2		A team of highly trained and experienced communications professionals manage communication during peacetime and emergencies.	Singapore
1-2-3		The MOH Communications and Engagement Group has sufficient human resources and funding and can access surge capacity from MCI.	Singapore
1-2-4		The system for risk communications is fully funded, covers surge capacity and is managed by trained communication officers.	Malaysia

1-2-5		There are dedicated staff members and financial resources for this area.	Oman
1-2-6		There is ongoing funding for communications staff and additional resources available in emergencies.	Australia
1-2-7		There is a dedicated team in the Health Portfolio to build risk communication capacity and emergency preparedness function among communications and policy/programme staff.	Canada
1-2-8		Members of the core team of communication specialists regularly undertake training and receive upto-date information on the risk communication procedures, including cooperation with line ministries.	Kyrgyzstan
1-2-9		Sufficient professional communication staff are available throughout the Swiss federal departments.	Switzerland
1-2-10		Communication capacity exists within other ministries. During a public health emergency, cross-sector collaboration occurs under the guidance of MOH Public Health Directorate and communications staff are repurposed as surge capacity, if needed.	Bahrain
1-2-11		The system enables risk communication surges of human and financial capacity in case of an emergency.	Belgium
1-2-12		Trained communication teams (public information officers, liaison officers and public relations officers) manage communication during non-emergency times and peacetime, give warnings prior to disasters, disseminate information during disasters, and provide advice during recovery periods.	Brunei Darussalam
1-2-13		Public relations officers within the Corporate Communications Unit at the MOH and/or other operations centre staff can be used as surge staff for risk communication during emergencies.	Brunei Darussalam
1-2-14		There a dedicated budget line for communication personnel, materials and activities for emergencies.	Palau
1-2-15		There are communications personnel and departments that formally respond to public information needs during emergencies.	Palau
1-2-16		Although this mechanism has not yet been tested, the national HCC could be used to mobilize risk communication surge staff to support response agencies during emergencies by sourcing risk communicators from agencies not involved in the response.	Palau
1-3-1	Roles and Responsibilities	There are several stakeholders with defined roles and responsibilities from government agencies, nonprofit organizations and the private sector that participate in the Media and Public Communication Emergency Supporting Plan.	UAE
1-3-2		Roles and responsibilities for risk communication are included in emergency response plans.	Canada
1-3-3		There are clear lines of responsibility among F/P/T and local officials for communications during emergencies.	Canada
1-3-4		The authority leading operations during a crisis also leads and coordinates communications. For largescale, multisectoral crises, responsibility can be raised to the ministerial level.	Finland
1-3-5		The lead agency's EOC PIO coordinates all risk communication activities in any relevant situation that has not been declared a national emergency.	Palau

1-4-1	Shared Communication Plans and SOPs	Plans and their effectiveness are tested periodically through joint drills among government agencies.	UAE
1-4-2		Systems are tested regularly in exercises and emergencies, and lessons learned are applied to strengthen capacity.	Australia
1-4-3		Best practices are shared between federal departments and with the cantons.	Switzerland
1-4-4		A Directorate, within the NCDM, led by the Undersecretary of the MOH, is being formalized to coordinate all public health and risk communication processes, linking them to other sectors during a disaster or an emergency.	Bahrain
1-4-5		Communications plans are applied in real life events and updated accordingly.	Brunei Darussalam
1-4-6		Risk communication has been integrated into simulation exercises and has been functional in recent health emergencies, namely, those relating to mass casualty and epidemic-prone disease events.	Kuwait
1-4-7		There is a well-established mechanism at the central government composed of inter-ministerial committees for gathering and disseminating information.	Tajikistan
1-5-1	Financial Resources for Emergencies	The MOH Communications and Engagement Group has sufficient human resources and funding and can access surge capacity from MCI.	Singapore
1-5-2		The system for risk communications is fully funded, covers surge capacity and is managed by trained communication officers.	Malaysia
1-5-3		There are dedicated staff members and financial resources for this area.	Oman
1-5-4		There is ongoing funding for communications staff and additional resources available in emergencies.	Australia
1-5-5		An emergency budget (5%) is set aside in each ministry, which includes financial resources for risk communications.	Bahrain
1-5-6		The system enables risk communication surges of human and financial capacity in case of an emergency.	Belgium
1-5-7		There a dedicated budget line for communication personnel, materials and activities for emergencies.	Palau
1-5-8		Following the MERS outbreak in 2015 and the reform of the National Disease Control system, the Office of Communication in KCDC was established with a trained workforce, budget, guidelines, and procedures to manage risk communication during public health emergencies.	Republic of Korea
1-6-1	Regular Testing and Training	Continuous improvements are made in the response plans of MHP and other agencies and authorities based on experience and outcomes of practical exercises, drills and actual responses. Such a mechanism also exists through lessons learnt from exercises carried out under NCEMA. Communications response staff are made aware of the updates and trained to accommodate them through regular workshops convened by NCEMA.	UAE
1-6-2		Regular exercises are conducted. The latest drill by MES on 19–20 July 2016 was on the establishment and functioning of an intersectoral joint information center in case of an emergency (major earthquake) with participation of representatives from government agencies.	Armenia

1-6-3		Communication protocols are regularly tested in exercises and used in real-world events.	Singapore
1-6-4		There are systems and processes for multisectoral coordination for risk communication that are regularly tested in simulation exercises and real events, with lessons applied to planning future communications.	Malaysia
1-6-5		Systems are tested regularly in exercises and emergencies, and lessons learned are applied to strengthen capacity.	Australia
1-6-6		Systems are tested regularly in exercises; lessons are learned and shared among all levels.	Canada
1-6-7		Members of the core team of communication specialists regularly undertake training and receive upto-date information on the risk communication procedures, including cooperation with line ministries.	Kyrgyzstan
1-6-8		Local information about potential threats and follow-up actions is used as training material for the specialist communication teams.	Kyrgyzstan
1-6-9		Communication staff are trained, procedures are regularly tested, and plans are evaluated in coordination with relevant sectors and levels; lessons are fed back into strategies.	Belgium
1-6-10		Communications training is regular for staff working on radiation and nuclear safety and there are joint national guidelines for managing radiation situations that include guidance on risk communications. Regular joint exercises are also conducted.	Finland
1-6-11		Agencies responding to emergencies take a common approach to risk communication planning and operations through the NEOC, and risk communication is included in agency and multiagency emergency exercises. Training in risk communication is conducted annually for emergency responders.	Palau
1-6-12		Communication mechanisms are tested on an annual basis during drills and actual events.	Palau
1-6-13		Risk communication has been integrated into simulation exercises and has been functional in recent health emergencies, namely, those relating to mass casualty and epidemic-prone disease events.	Kuwait
1-7-1	Messaging Clearance	Multiple crisis and risk communication media, tools, plans and templates.	Thailand
1-7-2		Key messages and communications channels are developed for different target groups.	Thailand
1-7-3		Multiple crisis and risk communication tools and templates.	USA
1-7-4		Accuracy is encouraged with “key message” documents, syndication of web content and coordination calls.	USA
1-7-5		Timeliness of risk communications is supported by pre-existing templates and tools that can be rapidly adapted to communication needs (e.g. templates for a first announcement of an outbreak).	Palau
1-7-6		The revised Infectious Disease Control and Prevention Act facilitates timely information disclosure that enhances rumour management in times of disease emergencies.	Republic of Korea

R.5.2 Internal and inter-partner communication and coordination

2-1-1	Coordination of Communication	Coordination and communication with partner organizations are tested yearly through NCEMA exercises and drills with participating agencies.	UAE
2-1-2		Communication is coordinated among crisis management centres and public relations units of MES, MoH and the Ministry of Territorial Administration (under which health facilities are organized).	Armenia
2-1-3		Processes and protocols with clearly defined communication roles and responsibilities are regularly tested and applied in emergencies.	Singapore
2-1-4		Communication and coordination protocols have been tested in real events and through exercises.	Malaysia
2-1-5		Some formal and informal mechanisms enable coordinated communication between stakeholders and partners, as well as the overall health sector.	Oman
2-1-6		NHEMRN coordinates risk communication among sectors, jurisdictions and stakeholders, jointly developing and sharing communication plans and products.	Australia
2-1-7		There are strong communications networks in place to coordinate public communications and risk communications, at all levels.	Canada
2-1-8		All internal partners and external stakeholders are invited to participate in joint meetings on risk communication matters to create an atmosphere of transparency and improve coordination.	Kyrgyzstan
2-1-9		During an emergency event, joint communication plans are developed in coordination with the relevant government sectors.	Bahrain
2-1-10		Brunei Darussalam has a whole-of-nation risk communications structure to respond promptly to health emergencies.	Brunei Darussalam
2-1-11		National actors have the ability and structures to coordinate communication effectively during domestic crisis, with clearly defined roles in some cases.	Finland
2-1-12		The respective functions of ministries, departments, agencies and other stakeholders are clearly defined, which made it easy to form a multisectoral working group for risk communications. This working group created a scaled up national plague risk communication plan which was collectively implemented by all members of the working group.	Seychelles
2-1-13		Several multisectoral high-level committees allow good coordination between stakeholders, in particular with the Ministry of Agriculture and the Ministry of Interior to address zoonosis or food safety matters.	Morocco
2-1-14		Recent responses to regional and national emergencies have tested interagency coordination of communications. DHBs regularly carry out and participate in exercises that include coordination and testing of communications with partner organizations.	New Zealand
2-1-15		During public health emergencies, the EpiNet team conducts weekly multi-sectoral updates to share information and respond to risk communication needs.	Marshall Islands
2-2-1	Stakeholders and Partner Agencies	Coordination and communication with partner organizations are tested yearly through NCEMA exercises and drills with participating agencies.	UAE
2-2-2		During drills, health care workers, media actors and national and international nongovernmental organisations, are active partners in the exercise as well as in adapting plans from lessons learned.	Armenia

2-2-3		Some formal and informal mechanisms enable coordinated communication between stakeholders and partners, as well as the overall health sector.	Oman
2-2-4		NHEMRN coordinates risk communication among sectors, jurisdictions and stakeholders, jointly developing and sharing communication plans and products.	Australia
2-2-5		Engagement with specific communities to develop appropriate messages for this audience (First Nations, for example).	Canada
2-2-6		A standardized mechanism has been introduced for the regular involvement of internal and external partners on communication issues.	Kyrgyzstan
2-2-7		Well planned and professionally organised internal communication procedures exist at federal level.	Switzerland
2-2-8		Thailand has informally and formally coordinated communication with all agencies under the Ministry of Public Health and the Department of Disease Control during an emergency.	Thailand
2-2-9		During an emergency event, joint communication plans are developed in coordination with the relevant government sectors.	Bahrain
2-2-10		There has been fruitful collaboration with the Institute for Tropical Medicine, Brussels Airlines and Brussels airport on the Ebola response.	Belgium
2-2-11		First line responders and media houses have been trained in risk communications.	Seychelles
2-2-12		There is a formal mechanism to coordinate internal communication within the MOH before, during and after an emergency. Recent responses to national and regional emergencies (such as the spread of dengue in the Pacific) have tested interagency coordination of communications.	Palau
2-3-1	Consistency of Information	The NICCL is an established network of lead communicators among the federal departments and agencies that serves to speak with a unified voice and consistent message.	USA
2-3-2		The success of Bahrain's internal and partner communications coordination is in part due to dynamic relationships and well-established informal processes, endorsements and agreements within government sectors. This is a strength as long as these informal processes are reinforced and augmented by documented procedures that are tested, reviewed and systematized.	Bahrain
2-3-3		With international organizations on common messages, through the WHO Communication Network; the Health Security Committee (HSC) Communicators' Network; and ECDC National Focal Points for Communication.	Belgium
2-3-4		National actors have the ability and structures to coordinate communication effectively during domestic crisis, with clearly defined roles in some cases.	Finland
2-3-5		First line responders and media houses have been trained in risk communications.	Seychelles
2-4-1	Formal Mechanisms	There is a formal mechanism to coordinate communication among civil society organizations and the private sector through the national response framework, in addition to agreements signed by the NCEMA.	UAE
2-4-2		All risk communication planning that includes multistakeholders' roles and responsibilities are in place.	Armenia

2-4-3		There is a robust risk communication structure at all levels of Government to promptly respond to health emergencies.	Singapore
2-4-4		Some formal and informal mechanisms enable coordinated communication between stakeholders and partners, as well as the overall health sector.	Oman
2-4-5		Formally coordinated communications such as documents from the Emergency Operations Centre to the Director of the Offices of Disease Prevention and Control at the regional level concerning Middle East Respiratory Syndrome.	Thailand
2-4-6		With the private sector, media, hospitals, partners and civil society, through formal and informal mechanisms.	Belgium
2-4-7		Clearly defined communication roles and responsibilities are outlined in the NaSOP.	Brunei Darussalam
2-4-8		Formal and informal collaboration and partnerships are in place between the MOH and health professional representatives and syndicates, as well as with religious leaders.	Morocco
2-5-1	Coordination with Healthcare Sector	At the MHP, communication with hospitals (government and private) during an emergency is done through the main NCEMA Emergency, Crisis and Disaster Operations Centre as well as through control rooms in hospitals. The same process happens at the local health authorities' hospitals of HAAD and DHA. Moreover, coordination with private hospitals is the remit of the local health authorities.	UAE
2-5-2		Some formal and informal mechanisms enable coordinated communication between stakeholders and partners, as well as the overall health sector.	Oman
2-5-3		With the private sector, media, hospitals, partners and civil society, through formal and informal mechanisms.	Belgium
2-5-4		Within the health and disability sector, the MOH is the hub that provides information to DHB communication teams, which are well-versed in messaging their communities through a range of networks and public information channels. DHBs are well connected within their communities and in turn provide intelligence back to the Ministry.	New Zealand
2-6-1	Coordination with Civil Society Organizations	There is a formal mechanism to coordinate communication among civil society organizations and the private sector through the national response framework, in addition to agreements signed by the NCEMA.	UAE
2-6-2		During emergencies, communication activities can also be coordinated with NGOs and the private sector.	Canada
2-6-3		With the private sector, media, hospitals, partners and civil society, through formal and informal mechanisms.	Belgium
2-6-4		There is involvement of multiple stakeholders and partners in information dissemination.	Mauritius
2-7-1	Testing Communication Coordination Exercises	Coordination and communication with partner organizations are tested yearly through NCEMA exercises and drills with participating agencies.	UAE
2-7-2		During drills, health care workers, media actors and national and international nongovernmental organizations, are active partners in the exercise as well as in adapting plans from lessons learned.	Armenia
2-7-3		Processes and protocols with clearly defined communication roles and responsibilities are regularly tested and applied in emergencies.	Singapore

2-7-4		Communication and coordination protocols have been tested in real events and through exercises.	Malaysia
2-7-5		Coordination is tested regularly in exercises and emergencies, and lessons learned are applied to strengthen capacity.	Australia
2-7-6		The Department of Disease Control and related departments have launched several awareness programmes such as hand washing, exercise, nutrition and well-being.	Thailand
R.5.3 Public communication			
3-1-1	Public Communication	All official agencies communicate with the public through Government communication departments, where dedicated teams are formally entrusted with media and social outreach. In case of emergency, communications with the public use many levels and methods, including regular media briefings and updates through mass communication, news, press conferences, television, as well as awareness and educational campaigns.	UAE
3-1-2		Malaysia uses a multitude of communication channels to reach audiences by maximizing traditional and social media, websites, public hotlines, printed materials, radio and mobile-based communication.	Malaysia
3-1-3		Policies and plans ensure that the government communicates about risks in times of emergencies in appropriate languages.	Canada
3-1-4		There are regular health behaviour campaigns, including pre-emergency preparedness messaging.	Indonesia
3-1-5		Collaborative arrangements are in place with public and private media which guarantees access for thedelivery of key risk communication messages.	Uganda
3-1-6		Media creating and offering space for health issues in their productions.	Mozambique
3-2-1	Media and Social Media Outreach	All official agencies communicate with the public through Government communication departments, where dedicated teams are formally entrusted with media and social outreach. In case of emergency, communications with the public use many levels and methods, including regular media briefings and updates through mass communication, news, press conferences, television, as well as awareness and educational campaigns.	UAE
3-2-2		The variety of communication platforms adopted can reach a wide audience to address public concern and rumours.	Singapore
3-2-3		The Health Education and Awareness programmes develop messages and materials for communication with a wide general public and different target audiences. This relies on the continuous engagement of the public and different media to reach large numbers of people and communities and to achieve comprehensive geographical coverage, in relevant languages. Different media channels used include social media and new technologies like SMS.	Oman
3-2-4		The MOH conducts social research to identify the best media and methods to disseminate risk communication messages and materials and guide their development. Cultural and language needs are considered, as well as which communication methods are best absorbed and internalised by the target audiences.	Palau
3-2-5		The KCDC has a state-of-the-art system for public communication that maximisesmaximises multiple platforms such as the media, social networks, the 24-hour KCDC Call Center (1339) and 1:1 text messaging that facilitates rapid and targeted communication.	Republic of Korea

3-2-6		There are established communications channels utilising both traditional and social media, such as newspapers, radio, websites, Twitter, YouTube and Facebook.	Japan
3-2-7		Dissemination of emergency information in local languages according to the needs of the audience (translation of key messages into 14 local languages) and the holding of media debates, community talks, community theatre and other events in local languages.	Angola
3-3-1	Target Audience Analysis	MOH has well-established links with community organisations for rapid information dissemination.	Singapore
3-3-2		Relevant local languages are used (largely Bahasa Indonesia, which is spoken nationally), as is English (especially in tourist areas).	Indonesia
3-3-3		The KCDC runs the Citizens' Communication Supporters and Experts' Communication Advisory to encourage the general public and stakeholders, respectively, to participate and engage in risk communication.	Republic of Korea
3-3-4		Carrying out CAP studies and media studies to determine whether a message reaches the target audience.	Angola
3-4-1	Training Public Spokesperson	There is a list of official and trained spokespersons in MHP and all health authorities, according to sectors of MHP. Messages must be cleared by the federal media agency (National Media Council) in coordination with NCEMA, which specifies what information can be delivered through public media.	UAE
3-4-2		The positive relationship with the media based on mutual trust helps during emergencies.	Singapore
3-4-3		Trained spokespeople have been identified at different levels of government.	Australia
3-4-4		Thailand's designated risk communicators and officers at all levels are trained in risk communications. At the national level, the Prime Minister or Government Spokesperson will give press interviews on health issues, for example on the Zika situation or Ebola preparedness.	Thailand
3-4-5		A trained communications team manages all aspects of public communication and engagement.	Brunei Darussalam
3-4-6		The Ministry of Health has a communications and press office that acts as a qualified public spokesperson.	Angola
3-5-1	Media Research	Pre- and post- testing of risk communication campaigns, to assess impacts in targeted groups.	Belgium
3-5-2		Collaborative arrangements are in place with public and private media which guarantees access for thedelivery of key risk communication messages.	Uganda
3-5-3		Media creating and offering space for health issues in their productions.	Mozambique
3-5-4		Carrying out CAP studies and media studies to determine whether a message reaches the target audience.	Angola
3-6-1	Message Adaptation	Messages are developed in languages relevant to the many ethnicities in Malaysia.	Malaysia
3-6-2		Relevant local languages are used (largely Bahasa Indonesia, which is spoken nationally), as is English (especially in tourist areas).	Indonesia

3-6-3		Communication materials are regularly developed and translated into several local languages.	Ethiopia
3-6-4		Efforts to adapt messages to specific targets are made; languages (French, Arabic and Amazigh), vocabulary, behaviours, etc.	Morocco
3-6-5		Dissemination of emergency information in local languages according to the needs of the audience (translation of key messages into 14 local languages) and the holding of media debates, community talks, community theatre and other events in local languages.	Angola
3-7-1	Regular Media Briefings	All official agencies communicate with the public through Government communication departments, where dedicated teams are formally entrusted with media and social outreach. In case of emergency, communications with the public use many levels and methods, including regular media briefings and updates through mass communication, news, press conferences, television, as well as awareness and educational campaigns.	UAE
3-7-2		Policies and plans ensure that the government communicates about risks in times of emergencies in appropriate languages.	Canada
3-7-3		The MOH Bureau of Communications and Public Service holds a regular weekly media briefing.	Indonesia
3-7-4		Engagement with the media is prioritized and takes place regularly.	Slovenia
R.5.4 Involvement in communication in areas affected by emergencies			
4-1-1	Social Mobilization	During emergencies, more robust and intensive community engagement measures are administered, including deployment of Grassroots Leaders and trained Dengue Prevention Volunteers to engage atrisk communities.	Singapore
4-1-2		An innovative approach of organizing volunteers for community engagement through the COMBI teams at national, state and district levels.	Malaysia
4-1-3		People/public/private partnerships (PPPP) play an important role in supporting the dissemination of information, education and communication (IEC) materials to the community	Indonesia
4-1-4		Social mobilization, behaviour change communication and community engagement take place regularly during an outbreak, and there is a well-established system of involving volunteers from the affected communities.	Albania
4-1-5		Teams or working groups can be developed ad hoc during an emergency to focus on social mobilization, health promotion or community engagement as needed during a response.	Montenegro
4-1-6		Members include community-based organizations and religious groups.	Eritrea
4-2-1	Community Engagement	Both Rapid Response Teams and the Health Education and Promotion Department conduct awareness events and campaigns in collaboration with Medical Districts, e.g. World Health Day campaigns.	UAE
4-2-2		The national response plan includes social mobilization and community engagement.	Singapore
4-2-3		There are a number of community engagement activities at different levels, and a fully operational area of health promotion and education in central and governorates, which coordinates the activities of social engagement throughout the country.	Oman

4-2-4		All levels of government engage communities across a broad range of public health topics and can mobilize them in an emergency.	Canada
4-2-5		Village health committees include community representatives and hold regular meetings.	Kyrgyzstan
4-2-6		There are MOUs in place with several community organizations, NGOs, corporations, community leaders, and religious leaders, to support community empowerment and health development. This also assists with disseminating messages and helps with the incorporation of local perspectives/input into developing and refining public messages and materials.	Indonesia
4-2-7		Feedback from local communities is achieved via the EBS unit and toll free line at the PHEOC, social media, tweet deck and via the districts.	Uganda
4-2-8		Social mobilization, health promotion and community engagement are included in national protection and rescue plans.	Montenegro
4-2-9		Senegal has a very dense network of associations, particularly at the community level (traditional communicators, representatives, neighbourhood sponsors, women's groups, sports and cultural associations, religious leaders). They are involved in communication activities.	Senegal
4-3-1	Health Promotion	Health promotion is implemented by regional and local MOH health facilities.	Armenia
4-3-2		There are a number of community engagement activities at different levels, and a fully operational area of health promotion and education in central and governorates, which coordinates the activities of social engagement throughout the country.	Oman
4-3-3		Health promotion specialists and volunteers are regularly trained and their skills updated.	Bahrain
4-3-4		Health promotion has both ongoing routine health care (addressing noncommunicable diseases and lifestyle issues) and emergency plans (disease outbreaks and other risks).	Bahrain
4-3-5		Social mobilization, health promotion and community engagement are included in national protection and rescue plans.	Montenegro
4-3-6		Health promotion annual review meetings are used as opportunities to share experiences among stakeholders (ministries of health, education, tourism, labour and human welfare, information; civil society organizations (such as National Union of Eritrean Youth and Students (NUEYS), National Union of Eritrean Women (NUEW), National Confederation of Eritrean Workers (NCEW)); and health promotion officers.	Eritrea
4-3-7		SNEIPS, with its regional and departmental subsidiaries, promotes and coordinates communication activities.	Senegal
4-4-1	Intermediate Level Functions	Health promotion is implemented by regional and local MOH health facilities.	Armenia
4-4-2		There are a number of community engagement activities at different levels, and a fully operational area of health promotion and education in central and governorates, which coordinates the activities of social engagement throughout the country.	Oman

4-4-3		Local community teams build relationships and networks that enable community action.	Canada
4-4-4		Village health committees include community representatives and hold regular meetings.	Kyrgyzstan
4-4-5		Representatives on village committees are trusted and serve as a point of connection for sending and receiving first-hand information on health issues.	Kyrgyzstan
4-4-6		For migrant populations, there is strong coordination between the Ministry of Labour and Social Affairs and MOH to provide health promotion materials and community engagement support in local languages and through health-care centres and religious leaders of these groups.	Bahrain
4-4-7		A clear structure exists for coordinating communications around multiple stakeholders.	Seychelles
4-4-8		There are MOUs in place with several community organizations, NGOs, corporations, community leaders, and religious leaders, to support community empowerment and health development. This also assists with disseminating messages and helps with the incorporation of local perspectives/input into developing and refining public messages and materials.	Indonesia
4-4-9		National and regional level health promotion working groups for outbreaks and health hazards exist.	Eritrea
4-5-1	Audience Feedback	Messaging is regularly and rapidly used to address audience feedback and questions, by detecting public reactions via different communication channels (i.e. call centres, social media, community outreach programmes, press conferences).	UAE
4-5-2		Feedback system from affected populations during the earthquake drills and Artik flood emergency was used to identify issues in need of improvement.	Armenia
4-5-3		Opinion polls to monitor public perceptions and reactions.	Thailand
4-5-4		There are MOUs in place with several community organizations, NGOs, corporations, community leaders, and religious leaders, to support community empowerment and health development. This also assists with disseminating messages and helps with the incorporation of local perspectives/input into developing and refining public messages and materials.	Indonesia
4-5-5		Feedback from local communities is achieved via the EBS unit and toll free line at the PHEOC, social media, tweet deck and via the districts.	Uganda
4-5-6		Refinement of messages has been carried out based on community feedback.	Uganda
4-5-7		Ministry of Information conducts ongoing random calls to test and receive feedback from the community.	Eritrea
4-6-1	Scaling Up Capacity	The 1422 hotline always has one officer available and can increase capacity to 30 officers during emergencies. Three English-speaking officers are available.	Thailand
4-6-2		There is regular briefing, training and engagement of social mobilization and community engagement teams, including volunteers. Mechanisms to scale up are operational.	Seychelles

4-6-3		Social mobilization, behaviour change communication and community engagement take place regularly during an outbreak, and there is a well-established system of involving volunteers from the affected communities.	Albania
4-6-4		Social mobilization, health promotion and community engagement are included in national protection and rescue plans.	Montenegro
4-6-5		Health promotion annual review meetings are used as opportunities to share experiences among stakeholders (ministries of health, education, tourism, labour and human welfare, information; civil society organizations (such as National Union of Eritrean Youth and Students (NUEYS), National Union of Eritrean Women (NUEW), National Confederation of Eritrean Workers (NCEW)); and health promotion officers.	Eritrea
4-7-1	Baseline Social Data	A recent survey of more than 10,000 participants was conducted on trusted information resources and preferred communication channels that will help to build better risk communication plans.	Armenia
4-7-2		There is an ongoing and functional feedback loop between vulnerable populations and response agencies.	Oman
R.5.5 Vigilant listening and rumour management			
5-1-1	Rumours and Misinformation	A formal communication function monitors and addresses rumours and misinformation through call centres and Government communication departments in MPH and local health authorities.	UAE
5-1-2		A system for daily monitoring and reporting on rumours is in place in the public relations department of the different ministries.	Armenia
5-1-3		The MOH research team conduct media surveillance and can rapidly respond to fake news and rumours, in conjunction with MOH decision makers.	Singapore
5-1-4		A system tracks and monitors rumours and misinformation from the public from different sources.	Oman
5-1-5		The public can convey information and complaints to the government through an integrated contact centre reachable through various channels: telephone, email, SMS, social media and the SIAP Kemenkes mobile app. All this constitutes the Saluran Informasi, Aspirasi, dan Pengaduan (SIAP) integrated information system.	Indonesia
5-1-6		The ROK has enhanced its capacity to rapidly communicate with the public to manage anxieties that can result from misinformation. This was evident when the first imported case of Zika virus infection was laboratory-confirmed in the ROK in March 2016. The KCDC immediately provided the public with accurate and easy-to-understand information, which was disseminated using various materials such as a visualized leaflet, videos, and card-news to dispel rumours regarding Zika virus infection and its mode of transmission.	Republic of Korea
5-1-7		Rumours are tracked through monitoring the emergency public hotline at the CDCU.	Mauritius
5-1-8		Rumours and misinformation are dealt with in a systematic and stepwise manner.	Mauritius
5-1-9		South Africa monitors rumours on social media and through rumour control coordinators, public information officers and community members.	South Africa
5-1-10		The country has a strong system for managing rumours.	Zambia

5-1-11		A media clipping service provides monitoring of the media to detect and guide responses to misinformation, rumours and risky behaviours.	Serbia
5-2-1	Monitoring Effectiveness	Social media sites are both scanned regularly and used to communicate messages.	UAE
5-2-2		Reports on double-checked rumours/information and communication strategies to counterbalance them, if necessary, are cleared in the management of each government department and shared between government bodies if required.	Armenia
5-2-3		Established SOPs provide strategic guidance to public healthcare institution responses to rumours and fake news.	Singapore
5-2-4		Traditional media and social media are monitored in emergencies to correct errors, address misconceptions and gauge opinion.	Australia
5-2-5		SOPs are in place to guide actions for addressing rumours and misinformation.	Indonesia
5-2-6		Rumours and misinformation are dealt with in a systematic and stepwise manner.	Mauritius
5-2-7		A media clipping service provides monitoring of the media to detect and guide responses to misinformation, rumours and risky behaviours.	Serbia
5-3-1	Feedback Loop	A coordination mechanism exists among all concerned through periodic meetings.	Oman
5-3-2		Village health committees are used as the main channel for verifying rumours and communicating risks.	Kyrgyzstan
5-3-3		The 1422 hotline is an effective channel both for providing information and addressing rumours and misinformation.	Thailand
5-3-4		Communications channels that accommodate public information, suggestions and complaints are mandated by Law No. 14/2008 on Public Information Transparency.	Indonesia
5-3-5		Rumours are addressed with correct information in local districts as well as door-to-door information and through drama.	Zambia
5-4-1	Public Health Issues	Lessons have been learnt from events, and behaviour change has been evidenced, for instance in managing rumours linking autism and MMR.	UAE
5-4-2		Health-related rumours rarely gain traction among the general public. In rural areas, the advice of health officials is considered to be authoritative.	Sri Lanka
5-4-3		The Ministry of Informatics and Communications (https://kominfo.go.id) also acts to counter hoaxes on social media through a digital literacy campaign programme and community initiative that provides search tools to check hoaxes: the TURNBACKHOAX.ID website; the TURNBACKHOAX mobile application; and the jpp.go.id website (@IndonesiaBaikId).	Indonesia
5-4-4		The ROK has enhanced its capacity to rapidly communicate with the public to manage anxieties that can result from misinformation. This was evident when the first imported case of Zika virus infection was laboratory-confirmed in the ROK in March 2016. The KCDC immediately provided the public with accurate and easy-to-understand	Republic of Korea

		information, which was disseminated using various materials such as a visualized leaflet, videos, and card-news to dispel rumours regarding Zika virus infection and its mode of transmission.	
5-5-1	Message Consistency	Reports on double-checked rumours/information and communication strategies to counterbalance them, if necessary, are cleared in the management of each government department and shared between government bodies if required.	Armenia
5-5-2		The management of risk communication is effective and the public trusts the MoH advice and recommendations.	Oman
5-5-3		By monitoring media and trends in public concerns, communications departments and agencies can address information gaps.	Finland
5-6-1	Evaluation of Communication Response	The communication response and ability to address rumours and misinformation is regularly evaluated to ensure that actions change behaviour and stop rumours from spreading.	UAE
5-6-2		The whole-of-government commination network allows agencies to dispel misinformation on a variety of platforms.	Singapore
5-6-3		Rumours are evaluated weekly with communication and technical experts, based on which it is decided whether and how to respond.	Oman
5-6-4		Communication responses—including the ability effectively to address perceptions, risky behaviour and misinformation—are evaluated regularly.	Brunei Darussalam
5-7-1	Behavioural Change	The communication response and ability to address rumours and misinformation is regularly evaluated to ensure that actions change behaviour and stop rumours from spreading.	UAE
5-7-2		Lessons have been learnt from events, and behaviour change has been evidenced, for instance in managing rumours linking autism and MMR.	UAE
5-7-3		Communication responses—including the ability effectively to address perceptions, risky behaviour and misinformation—are evaluated regularly.	Brunei Darussalam
5-7-4		Rumours and misinformation are dealt with in a systematic and stepwise manner.	Mauritius
